# Targeted Intraoperative Radiotherapy (TARGIT-IORT) for Early-Stage Invasive Breast Cancer: A Single Institution Experience

**DOI:** 10.3389/fonc.2022.788213

**Published:** 2022-06-29

**Authors:** Andrea Brown, Elizabeth J. Buss, Christine Chin, Gaotong Liu, Shing Lee, Roshni Rao, Brett Taback, Lisa Wiechmann, David Horowitz, Julie C. Choi, Leah M. Katz, Eileen P. Connolly

**Affiliations:** ^1^ Department of Radiation Oncology, Columbia University Irving Medical Center, New York, NY, United States; ^2^ Department of Biostatistics, Columbia University Mailman School of Public Health, New York, NY, United States; ^3^ Department of Surgery, Columbia University Irving Medical Center, New York, NY, United States

**Keywords:** breast cancer, intraoperative radiotherapy (IORT), partial breast radiotherapy (PBI), radiation treatment, breast surgery

## Abstract

**Purpose/Objective:**

We present our single-institution experience in the management of invasive breast cancer with targeted intraoperative radiotherapy (TARGIT-IORT), focusing on patient suitability for IORT determined by the American Society for Radiation Oncology (ASTRO) Accelerated Partial Breast Irradiation (APBI) consensus guidelines.

**Materials/Methods:**

We identified 237 patients treated for biopsy-proven early-stage invasive breast cancer using low energy x-ray TARGIT-IORT at the time of lumpectomy between September 2013 and April 2020 who were prospectively enrolled in an institutional review board (IRB) approved database. We retrospectively reviewed preoperative and postoperative clinicopathologic factors to determine each patient’s ASTRO APBI suitability (suitable, cautionary or unsuitable) according to the 2017 consensus guidelines (CG). Change in suitability group was determined based on final pathology. Kaplan-Meier methods were used to estimate the survival probability and recurrence probability across time.

**Results:**

237 patients were included in this analysis, based on preoperative clinicopathologic characteristics, 191 (80.6%) patients were suitable, 46 (19.4%) were cautionary and none were deemed unsuitable. Suitability classification changed in 95 (40%) patients based on final pathology from lumpectomy. Increasing preoperative lesion size or a body mass index (BMI) ≥ 30 kg/m^2^ were significant predictors for suitability group change. Forty-one (17.3%) patients received additional adjuvant whole breast radiotherapy after TARGIT-IORT. At a median follow up of 38.2 months (range 0.4 – 74.5), five (2.1%) patients had ipsilateral breast tumor recurrences (IBTR), including two (0.8%) true local recurrences defined as a recurrence in the same quadrant as the initial lumpectomy bed with the same histology as the initial tumor. IBTR occurred in 1/103 (0.09%) patient in the post-op suitable group, 4/98 (4.08%) patients in the post-op cautionary group, and no patients in the post-op unsuitable group. At 3-years, the overall survival rate was 98.4% and the local recurrence free survival rate was 97.1%.

**Conclusion:**

There is a low rate of IBTR after TARGIT-IORT when used in appropriately selected patients. Change in suitability classification pre to postoperatively is common, highlighting a need for further investigation to optimize preoperative patient risk stratification in this setting. Patients who become cautionary or unsuitable based on final pathology should be considered for additional adjuvant therapy.

## Introduction

Breast cancer is the most common female malignancy diagnosed in the United States, with 67% of cases detected at stage 1 or 2 (1). Currently, the standard of care for early-stage breast cancer is breast conserving surgery followed by adjuvant whole breast irradiation (WBI) or mastectomy alone. Accelerated partial breast irradiation (APBI) was developed as a type of radiotherapy de-escalation for patients with a low risk of recurrence. The concept of APBI is based on evidence that most local in-breast recurrences after breast conservation therapy occur in the breast tissue adjacent to the lumpectomy cavity (2). Intraoperative radiation therapy is one of several forms of APBI. TARGIT-IORT delivers a single fraction of radiation directly to the lumpectomy cavity at the time of surgery, providing an attractive treatment option for patients as it is both time and cost effective (3, 4). Two large phase III randomized trials have compared WBI with IORT using either electrons (ELIOT) or low-kilovoltage x-rays (TARGIT-A) (4, 5).

The ELIOT trial compared IORT using electrons versus WBI with a boost to the lumpectomy cavity, showing initially that IORT resulted in a 5-year ipsilateral breast tumor recurrence (IBTR) risk of 4.4% (35/651) compared to a risk of 0.4% (4/654) for conventional WBI (5). The ELIOT trial included patients with a higher risk profile, with 21% of patients enrolled having N1 disease. Notably, it was found that with additional patient risk stratification, those patients who fit the ASTRO APBI consensus guidelines (CG) suitable criteria had a 5-year occurrence of IBTR of 1.5% (6). The recently published long-term recurrence and survival outcomes from the ELIOT trial report a significantly higher occurrence of IBTR in patients allocated to the ELIOT group than in those in the WBI group, with a 15-year IBTR rate of 12.6% in those patients treated with IORT and 2.4% for those treated with conventional WBI (7). Again, however, additional risk stratification of patients identified a subgroup of patients with a very low risk of IBTR that represented 10.8% of the study population, consisting of patients with a tumor size < 1 cm, a grade 1 tumor, luminal A molecular subtype, and a proliferative index (Ki-67) < 14%. The incidence of IBTR in this subgroup was 1.3% at 10 years (7). The 10-year cumulative incidence of IBTR in patients assigned to a suitable group according to ASTRO APBI CG was 6.1%, highlighting the significance of patient selection when IORT is the chosen treatment modality (8).

The TARGIT-A trial compared IORT using low-energy photons (Zeiss Interbeam system) versus WBI; the study included two groups, those receiving TARGIT-IORT at the time of initial resection (immediate group) and those who received delayed TARGIT-IORT as a second procedure (delayed group). The initial outcomes were reported for the entire study population (immediate and delayed groups) and demonstrated a 5-year IBTR risk of 3.3% for TARGIT-IORT and 1.3% for WBI, establishing at that time non-inferiority within the study’s predetermined non-inferiority margin (9). The TARGIT-IORT eligibility was not confined to low-risk patients however patients needed to be 45 years or older, with invasive ductal carcinoma that was suitable for breast conservation, less than 3.5 cm in size and unifocal on clinical examination and conventional imaging (9, 10). Approximately 20% of patients assigned to the immediate TARGIT-IORT arm also received adjuvant WBI in a risk-adapted schema based on surgical pathology results. Long-term outcomes of the TARGIT-A trial show that after a median follow up of 8.6 years, 5-year local recurrence risk in the immediate group for the TARGIT-IORT arm was 2.11% versus 0.95% in the WBI arm (10). Results for the delayed group were reported separately and showed increased local recurrence with TARGIT-IORT at 5-years (3.96% TARGIT-IORT vs. 1.05% WBI) that exceeded the noninferiority threshold (11).

The appropriate use of TARGIT-IORT remains controversial and while these studies demonstrated higher rates of recurrence with delayed IORT, there is a role for immediate TARGIT-IORT in most patients suitable for breast conserving surgery. At our institution, patients pre-operatively deemed to be suitable or cautionary by the 2017 ASTRO APBI CG are offered low-energy x-ray TARGIT-IORT after multidisciplinary evaluation. The present analysis reports the clinical outcomes of these patients.

TARGIT-IORT presents a unique challenge in risk stratification by ASTRO APBI CG because tumor characterization in the preoperative period may differ from final pathology determined postoperatively, thus changing patient suitability classification after the procedure has been performed. While recent clinical and biological subgroup analysis of the TARGIT-A trial suggests that local recurrence-free survival between the IORT and EBRT arms is equivalent irrespective of tumor size, grade, estrogen/progesterone/HER2 receptor status and/or lymph node status, other clinical patient factors such as age, breast density and/or body mass index (BMI) measurements may also influence the probability of reclassification due to discrepancies in clinical and pathologic tumor size, margins, occult multifocal/centric disease that may ultimately lead to additional whole breast therapy (12). Limited data exists examining the preoperative patient and tumor clinicopathologic characteristics that are most likely to result in a change in suitability classification postoperatively. Herein, we present our single-institution experience with early-stage invasive breast cancer TARGIT-IORT, focusing on changes in group suitability classification determined by ASTRO 2017 APBI CG criteria. Other published single-institution experiences have reported DCIS and early-stage breast cancer TARGIT-IORT treatment outcomes with similar rates of local recurrence as reported in the ELIOT and TARGIT-A trials; however, we further aim to describe the preoperatively determined consensus guideline criteria, breast density classifications, and BMI measurements most likely to result in postoperative group suitability changes and subsequent need for adjuvant WBI (13, 14).

## Materials and Methods

### Patient Eligibility

Eligibility for IORT is initially determined by preoperative screening of patients by breast surgeons, patients must have a screen detected, unifocal lesions < 3cm in size, which is hormone receptor positive, HER2 negative and clinically node negative. Radiation oncology sees each patient for consideration of IORT and if there is agreement on eligibility, the patient was enrolled onto our IRB-approved institutional prospective registry database AAAJ8512. For this analysis, we retrospectively identified from within this registry patients with biopsy-proven invasive breast cancer treated with TARGIT-IORT using the Zeiss Intrabeam ^®^ device between September 2013 and April 2020. We excluded patients from analysis with prior ipsilateral breast irradiation or pure ductal carcinoma in situ. Patients were classified as suitable, cautionary, or unsuitable according to the 2017 ASTRO APBI CG based on preoperative clinicopathologic data and then reclassified in the postoperative period based on results of the final surgical pathology.

### Preoperative Imaging and Pathologic Evaluation

Preoperative assessment of lesion size for eligibility for TARGIT-IORT was based on conventional imaging using mammogram and/or ultrasound, and MRI for patients with discordant measurements. We determine patient breast density using the diagnostic mammogram report in which breast radiology classified patient’s breast as entirely fatty, scattered fibroglandular densities, heterogeneously or extremely dense as per standard guidelines (15). MRI was ordered at the discretion of the surgeon and was not required for TARGIT-IORT.

### Intraoperative Treatment

The Zeiss Intrabeam ^®^ device was used to deliver a dose of 20 Gy to the lumpectomy cavity at the time of breast conserving surgery using the TARGIT technique (9). A spherical applicator was chosen by the radiation oncologist and operating surgeon to appropriately fit the lumpectomy cavity (1.5-5.0cm), maintaining a skin-to-applicator distance of approximately 10 mm at 12, 3, 6, and 9 o’clock from the applicator surface, which was confirmed by real-time ultrasound. Sentinel lymph node biopsy was routinely performed at the time of lumpectomy. The final surgical pathology report was reviewed with patients at follow-up 2 weeks after surgery.

### Recommendations for Additional Therapy

Re-excision followed by adjuvant WBI (40.5 Gy- 42.56 Gy in 15-16 fractions) was recommended for patients with positive surgical margins. Additional WBI alone (40.5 Gy- 42.56 Gy in 15-16 fractions) was routinely discussed and strongly recommended in the presence of high-risk pathologic features which would cause the patient to be classified as cautionary or unsuitable for APBI alone according to the 2017 ASTRO APBI consensus guidelines (6). These include final pathologic lesion size >3 cm, close surgical margins of < 2 mm, lobular histology, extensive multifocal disease, lymphovascular invasion, occult nodal positivity, and occult multicentric disease.

### Endpoint and Follow-Up

We reviewed patient charts to determine incidence and characteristics of biopsy-proven locoregional recurrences, distant metastases, and survival, patients who had not been seen in person in past 6 months were contacted by phone call to confirm status. IBTR was defined as any new invasive or DCIS lesion in the index breast. True local recurrence was defined as an invasive lesion in the same quadrant as the initial lumpectomy bed with the same histology as the initial tumor. Overall survival was defined from the date of surgery to last follow-up. Recurrence free survival was also determined from date of surgery to last follow up. We documented patient compliance with recommended hormonal therapy at last follow-up. The details regarding any salvage therapy at the time of recurrence were recorded. Changes in patient preoperative and postoperative suitability classification were recorded.

### Statistical Analysis

Frequency with percentage or median with ranges were reported for baseline and clinical characteristics. Chi-squared test or Fisher’s exact test was used to compare the distribution of these variables by MRI status. Change in patient status was assessed as a binary outcome. A multivariable logistic regression model was used to assess the associations between age, BMI, breast density, MRI, preoperative grade, preoperative size, and each binary outcome. Kaplan-Meier estimator was used to estimate the survival probability and recurrence probability across time for all patients and Kaplan-Meier curves were generated accordingly. Three-year survival probability and recurrence probability were reported with 95% confidence interval. All analyses were conducted by R (version 3.6.3) with a significance level of 0.05.

## Results

### Patient Characteristics and Preoperative APBI Suitability as per 2017 ASTRO CG

There were 360 patients enrolled in the registry. Patients with DCIS and patients for whom TARGIT-IORT was planned as an upfront boost or for re-irradiation were excluded from analysis. In turn, there were 237 patients included in the analysis. The median patient age was 67 years (range 44-94 years). The median extent of disease was 1.0 cm (range 0 - 3.0cm). The majority of patients were disease subtype Luminal A (200/237, 84.4%), 35 (14.8%) were Luminal B (defined as HR+/HER2 neg with Ki67 >20%) and 2 (0.8%) were HER2 enriched. Median BMI of the patient cohort was 27.6 kg/m^2^, with 78 (33%) patients classified as obese (BMI ≥30 kg**/**m^2^) and 159 (67%) patients not obese (BMI <30 kg**/**m^2^). Breast density was classified as not dense if described on radiographic report as almost entirely fatty (15/237, 6.3%) or scattered fibroglandular densities (113/237, 47.7%), and was classified as dense if described as heterogeneously dense (100/237, 42.2%) or extremely dense (3/237, 1.3%). A summary of patient clinical and final pathologic characteristics can be found in **Table 1**. MRI was obtained in addition to conventional imaging in 102 (43%) patients. A univariate analysis of predictors of obtaining preoperative MRI can be seen in **Supplemental Table 1**. Factors that were found to be significant on multivariable analysis for whether a patient underwent preoperative MRI imaging were surgeon, BMI, and age, see **Table 2**. We found that patients with a BMI ≥ 30 kg/m^2^ or over the age of 60 were less likely to undergo MRI. Breast density was not significantly correlated with whether a patient underwent MRI. Notably, in our dataset, BMI was inversely correlated with breast density (p = 0.00066). Based on preoperative clinicopathologic characteristics, 191 (80.6%) patients were suitable according to ASTRO APBI guidelines, 46 (19.4%) were cautionary, and none were deemed unsuitable.

**Table 1:** Patient characteristics (n = 237).

Characteristics	n (%)
**Age**	
40-49 50-69 70+	10 (4.2) 138 (58.2) 89 (37.6)
**Body Mass Index**	
<30 ≥30	159 (67) 78 (33)
**Breast Density**	
Almost Entirely Fatty Scattered Fibroglandular Densities Heterogeneously Dense Extremely Dense Unknown	15 (6.3) 113 (47.7) 100 (42.2) 3 (1.3) 6 (2.5)
**Magnetic Resonance Imaging**	
Yes No	102 (43) 135 (57)
**Clinical Size**	
≤2 cm 2.1-3 cm	226 (95.4) 11 (4.6)
**Grade**	
Low Intermediate High Unknown	81 (34.2) 137 (57.8) 14 (5.9) 5 (2.1)
**Subtype**	
Luminal A Luminal B HER2 Enriched	200 (84.4) 35 (14.8) 2 (0.8)
**Invasive Margins**	
Positive <2 mm 2.1-2.9 mm ≥3 mm	15 (6.3) 20 (8.4) 21 (8.9) 181 (76.4)
**DCIS Margins**	
Positive Negative	7 (3%) 230 (97%)
**Postoperative Nodal Status**	
Positive (N1) Negative (N0)	15 (6%) 230 (94%)

**Table 2:** Multivariate analysis of predictors of obtaining preoperative MR.

Characteristics	Odds Ratio	95% Confidence Interval	p-value
**Age group**			0.0001
50-60 years	–	–	
60+ years	0.2517	0.1285, 0.4930	
**BMI**			0.0217
Not Obese (< 30)	–	–	
Obese (30+)	0.6421	0.4399, 0.9372	
**Breast Density on Mammogram**			0.5441
Fatty	–	–	
Dense	1.1994	0.6665, 2.1585	
**Surgeon**	0.7947	0.6809, 0.9275	0.0036

BMI, body mass index.

### Final Pathologic Finding and Changes in Patient APBI Suitability

**Figure 1:**
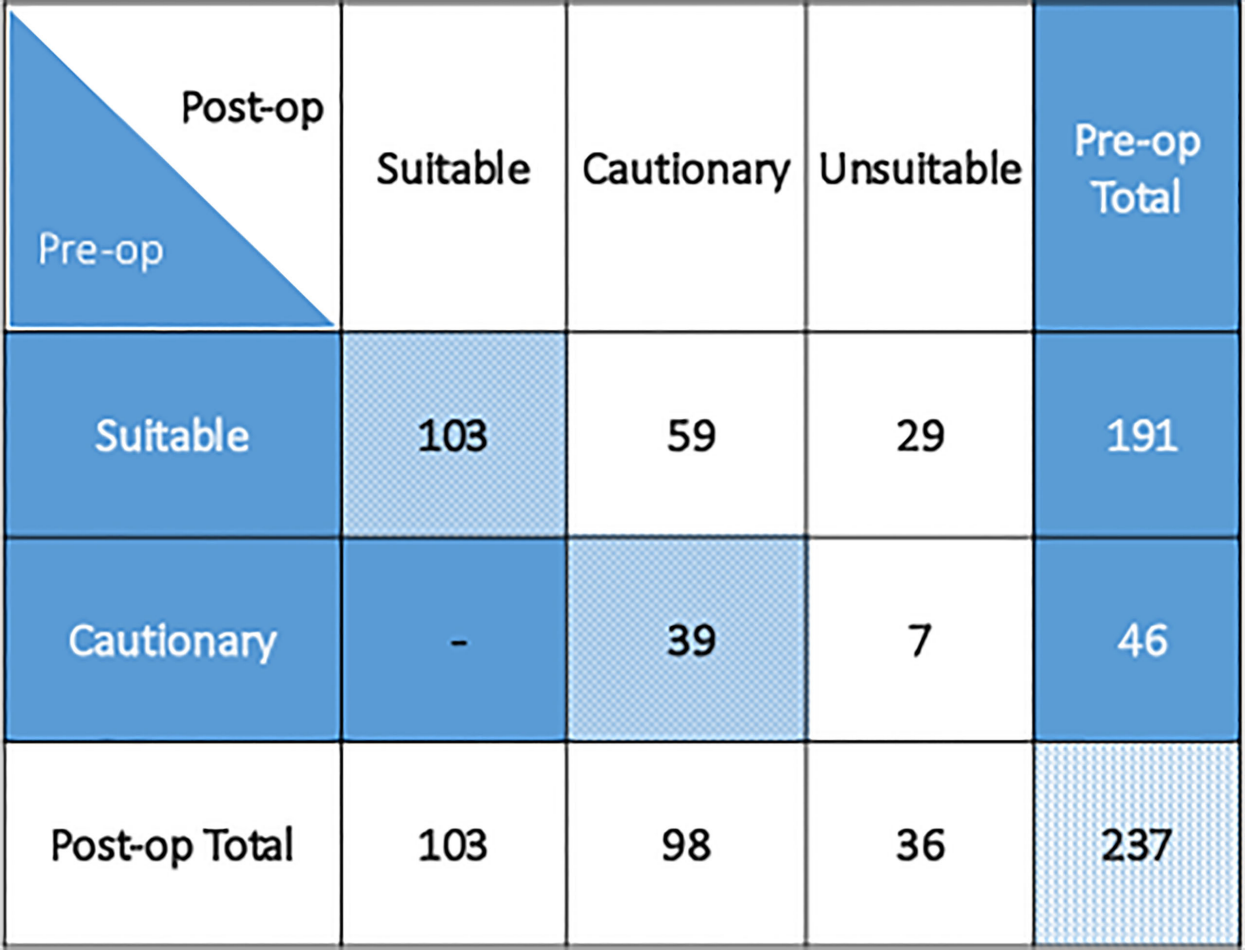
Patient preoperative and postoperative group suitability classification as per 2017 ASTRO APBI consensus guideline.

APBI CG suitability classification changed in 95 (40%) patients based on final pathology from the time of surgery, summarized in **Table 3** and illustrated in **Figure 1**. Median lesion size was 1.0 cm (range 0 – 3.0 cm). Sentinel node biopsy was performed in 221/237 (93.2%) patients.

**Table 3:** Criteria for Pre-Operative Suitability Classification and Post-Operative Suitability Change.

	Reason										
**Pre-Op Suitability**	#	Size	ER (-)	Age		ILC				
**Suitable**	191									
**Cautionary**	47	11	4	10		22				
**Unsuitable**	0									
**Post-Op Suitability Change**	#	Size	Close Margins	IDC/ILC(+) Margins	DCIS (+) Margins	LVI	ILC	EIC	Occult N(+)	Occult Multicentric
**Suitable to Cautionary**	59	8	18			13	10	19		
**Suitable to Unsuitable**	29	1		9	7				13	2
**Cautionary to Unsuitable**	7	2		3					2	

ER, Estrogen Receptor; ILC, Invasive Lobular Carcinoma; IDC, Invasive Ductal Carcinoma; DCIS, Ductal Carcinoma in Situ; LVI, Lymphovascular Invasion; EIC, Extensive Intraductal Component;, N, Nodal Disease.

Among the 191 patients classified preoperatively as suitable, 59 (31%) became cautionary due to high-risk features including lesion size (2.1-3.0 cm), close surgical margins (<2mm), invasive lobular histology, lymphovascular invasion, or extensive intraductal component. Twenty-nine (15%) initially suitable patients became unsuitable postoperatively due to lesion size (>3.0 cm), positive surgical margins, occult multicentric disease, or occult nodal positivity. Of 46 patients preoperatively determined to be cautionary, 7 (15%) patients became unsuitable postoperatively due to lesion size, positive surgical margins, or occult nodal positivity. Three out of 36 (8%) unsuitable patients were unsuitable for more than one reason.

To evaluate the preoperative factors predictive of suitability group change, we excluded 10 patients under the age of 50 and 11 patients with preoperative lesion size >2.0 cm because these factors are deterministic of patient suitability classification and cannot demonstrate group change. In a univariate analysis we found age, BMI, and preoperative MRI to be significant, see **Supplemental Table 2**.** **A multivariable analysis was then performed. In this analysis, we found that a BMI ≥ 30 kg/m^2^ or a larger preoperative lesion size are statistically significant preoperative predictors of postoperative suitability group change, see **Table 4**.

**Table 4:** Multivariate analysis of predictors of suitability group change.

Characteristics-	Odds Ratio	95% Confidence Interval	p-value
**Age group**			0.34
50 -64 years	–	–	
65+ years	1.350	0.725, 2.539	
**BMI**			0.05
Not Obese (< 30)	–	–	
Obese (30+)	1.883	0.994, 3.605	
**Breast Density on Mammogram**			0.24
Fatty	–	–	
Dense	1.442	0.785, 2.672	
**Preoperative MRI**			0.17
No	–	–	
Yes	0.647	0.343, 1.209	
**Preoperative Grade**			0.29
Low	–	–	
Intermediate	0.686	0.367, 1.275	
High	1.491	0.418, 5.449	
**Preoperative Size (continuous)**	2.948	1.474, 6.112	0.003

### Additional Therapy After TARGIT-IORT

Forty-one (17.3%) patients received additional adjuvant whole breast radiotherapy, including 30/36 (83%) of unsuitable patients. The mean dose of radiation prescribed for WBI was 40.5 Gy delivered in 15 daily fractions. Of the 12 patients deemed unsuitable due to positive invasive margins after surgery, 8 underwent re-excision with adjuvant WBI, 2 received adjuvant WBI alone, as they declined re-excision, and 2 declined additional surgery or radiation. Of the 7 patients with positive DCIS margins, 5 underwent re-excision with adjuvant WBI and 2 underwent re-excision alone, as they declined adjuvant WBI. There were 3 patients postoperatively classified as unsuitable due to lesion size; 1 received adjuvant WBI and 2 declined. Two patients had occult multicentric disease; 1 underwent total mastectomy and 1 received adjuvant WBI. Occult nodal disease was discovered in 15 patients all of which were pN1. Of these 15 cases; 2 underwent re-excision due to a positive margin followed by adjuvant WBI, 11 received adjuvant WBI alone, and 2 declined additional surgery and radiation. Patients with occult nodal disease were treated with WBI using high tangent fields to cover the low axillary lymph nodes, no patients received comprehensive nodal irradiation. One patient classified as cautionary underwent re-excision for close DCIS margins and 11 received adjuvant WBI for high grade or HER2+ disease.

**Figure 2:**
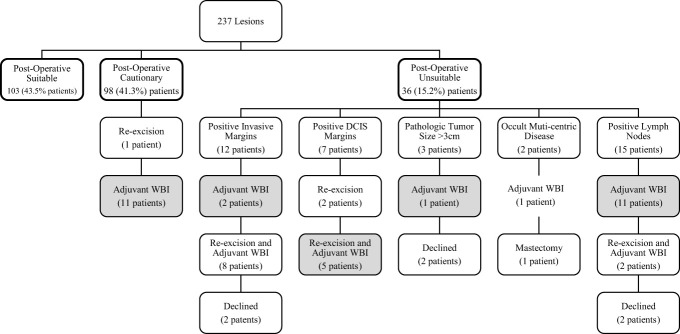
Patient post-operative ASTRO consensus guideline group suitability and additional therapy.

Hormone therapy was recommended to all women with hormone receptor-positive disease. Of 212 (89.5%) patients who initiated hormone therapy (HT), 191 (80.6%) remained compliant at last follow-up, with a median follow-up of approximately 3 years (range 0.4 - 74.5 months). Chemotherapy was prescribed for any reason in 25 patients, including 3/103 (2.9%) suitable patients, 9/98 (9.2%) cautionary patients, and 13/36 (36.1%) unsuitable patients, this was at the treating medical oncologists discretion. Adjuvant therapy corresponding to postoperative group suitability is summarized in **Figure 2**.

### Local Recurrences and Salvage Therapy

The median follow-up for our patient cohort was 38.2 months (range 0.4 - 74.5), with > 95% of patients having confirmed data within 6 months of analysis. Five (2.1%) patients had ipsilateral breast tumor recurrences, 1 (0.4%) patient had new contralateral breast cancer, and 2 (1.6%) had distant recurrences. Ipsilateral breast tumor recurrence occurred in 1/103 (0.09%) patient in the post-op suitable group, 4/98 (4.08%) patients in the post-op cautionary group, and no patients in the post-op unsuitable group. Of the 5 patients with IBTR, 2 (0.8%) were in the same quadrant as the initial lumpectomy bed with the same histology as initial tumor. Both of these true local recurrences were cautionary risk and one declined HT. There were 3 (1.3%) new breast primaries occurring outside the original quadrant with a different histology. Characteristics of patients with any tumor-related event are summarized in **Table 5**. At 3-years, the overall survival rate was 98.4%, 95%CI [96.47% - 100%] and the local recurrence free survival rate was 97.1%, 95%CI [95.56% -99.69%].

**Table 5:** Summary of events (n = 8).

Pre-op suitability	Post-op suitability	Subtype	High-risk features	HT	WBI	Recurrence Location	Time to event (months)	Salvage therapy	Initial Histology	Event Histology
Suitable	Cautionary	Luminal A	Size, LVI	Yes	No	IBTR (True)	12.2	Mastectomy	IDC	IDC
Cautionary	Cautionary	Luminal B	ER-, EIC	No	No	IBTR (True)	20.6	None	IDC	IDC
Suitable	Cautionary	Luminal A	EIC	No	No	IBTR	50.4	BCS, WBI	IDC	DCIS
Suitable	Suitable	Luminal A		Yes	No	IBTR	6.3	BCS, WBI	IDC	DCIS
Cautionary	Cautionary	Luminal A	ILC, margins	No	Yes	IBTR	47.6	Mastectomy	ILC	IDC
Cautionary	Unsuitable	Luminal A	margins	Yes	Yes	Contralateral breast	7.3	Bilateral mastectomy	IDC	IDC
Suitable	Cautionary	Luminal A	EIC	Yes	Yes	Distant metastases	44.1	Chemo	IDC	IDC
Cautionary	Cautionary	Luminal A	LVI, ILC	Yes	Yes	Distant metastases	35.0	Chemo	ILC	ILC

HT, hormonal therapy; WBI , whole breast irradiation; IBTR, ipsilateral breast tumor recurrence; True, True local recurrences located in the same quadrant as original tumor; LVI, lymphovascular invasion; IDC, invasive ductal carcinoma; ER, estrogen receptor; EIC, extensive intraductal component; BCS, breast conserving surgery; DCIS, ductal carcinoma in situ; ILC, invasive lobular carcinoma.

## Discussion

The results of this analysis show a low rate of local recurrence with the use of TARGIT-IORT in a large single intuition cohort of early breast cancer patients with a median age of 64. The 2.1% IBTR rate is similar to the TARGIT-A trial, which demonstrated a 2.11% rate of IBTR with a 5-year complete follow-up (9). This is despite 40% of patients having a change in their ASTRO APBI CG suitability group pre to post-operatively and consistent with more recent subgroup analysis from the TARGIT-A trial suggesting that there is no difference in the local control achieved by TARGIT-IORT and EBRT, irrespective of tumor size or subtype (12). Similar to the TARGIT-A trial 17.3% of patients received additional WBI, most of whom were classified as unsuitable postoperatively.

Our analysis is one of the first to explore ASTRO APBI CG suitability group change as a means of further refining preoperative patient selection for TARGIT-IORT. Our results demonstrate the limitations of the ASTRO APBI guidelines in selecting patients for TARGIT-IORT. Change in suitability classification pre to postoperatively was common, with 40% of patients in our cohort changing classification based on final pathology. Many of the factors that led to additional WBI in our experience were high-risk pathologic findings (margin status, occult lymph node involvement, extensive ductal carcinoma in situ) present within a largely clinically favorable patient tumor cohort thus highlighting the need to further optimize preoperative patient selection with less traditional clinical factors. The goal of examining the clinicopathologic factors predictive of suitability group change is to identify factors that may not inherently define a patient’s recurrence risk in the preoperative setting, but will predict for a high likelihood of finding occult high-risk pathologic factors in the postoperative period. In our analysis, we identify clinical factors, such as preoperative MRI, BMI, and breast density, that with further investigation may allow for additional refinement of preoperative patient risk to improve patient outcomes with TARGIT-IORT.

In our study, 43% patients underwent preoperative MRI in addition to conventional imaging. This is in contrast to 5.6% patients who underwent MRI in the TARGIT-A trial. In 2015, Tallet et al. published a single-institution study examining the impact of preoperative MRI on patient suitability for TARGIT-IORT. Of 175 patients, 138 (79%) underwent MRI during treatment workup (16). Ipsilateral lesions classified as ACR3 (probably benign) and ACR4 (suspicious) were detected by MRI in 33 (23%) of patients who then underwent a second ultrasound which classified 21 (15%) lesions as ACR5 (cancer) necessitating biopsy. In total, 7 (4%) patients were diagnosed with multifocal tumors and were deemed unsuitable for APBI. In our analysis, not undergoing an MRI was significantly predictive of suitability group change on univariate analysis but not in subsequent multivariable analysis. A BMI ≥ 30 kg/m^2^ or a larger preoperative lesion size were significant preoperative predictors of postoperative suitability group change on multivariable analysis. We found that women with BMI ≥ 30 kg/m^2^ were significantly less likely to undergo MRI and were more likely to be classified as having less dense breast tissue. In our cohort BMI was inversely correlated with breast density (p = 0.00066), and it is possible that BMI acted as a surrogate inverse measure of breast density.

To our knowledge, there is no data comparing BMI or breast density with group suitability classification. BMI is a valuable parameter in breast cancer patients because higher BMI has been shown to correlate with non-palpable tumors on physical exam, advanced stage disease at diagnosis, and increased risk for local recurrence after treatment (17). Breast density is a volumetric measurement of radiographically dense tissue in the breast and has been shown to correlate with an increased risk of developing breast cancer and decreased sensitivity of screening mammography (18). Reporting of breast density, however, is a subjective process, and therefore variable (15). Breasts may be described as almost entirely fatty, scattered fibroglandular densities, heterogeneously dense, or extremely dense in order of increasing breast density. Patients with dense breasts are more likely to have discordant preoperative lesion measurements *via* conventional ultrasound and mammogram imaging techniques, additional evaluation *via* MRI to confirm lesion size may be recommended in this subset of patients (19). Although obesity is negatively correlated with breast density, both are independent risk factors for decreased tumor detection on imaging (20). Interestingly, in our study, breast density was not significantly correlated with whether a patient underwent MRI. This may be, in part, due to the subjectivity of the breast density descriptor. Within our cohort, we found there was little variability in the breast density metric with the majority of patients grouped as either scattered fibroglandular or heterogeneously dense. Therefore, it is feasible that the higher rate of postoperative suitability group change seen in patients with BMI ≥ 30 kg/m^2^ is due to lower probability of these patients undergoing MRI imaging due to them being less likely to be classified as having dense breast tissue.

Further investigation into the role of BMI and breast density in the setting of TARGIT-IORT risk stratification, and the utility of MRI in relation to these clinical factors, is warranted. A more objective means of defining and reporting breast density is likely a critical component of any attempt to accurately relate these metrics. Additionally, given the ability of breast tissue composition to effect RBE values of low-energy x-rays during TARGIT-IORT, more objective breast density calculations may also allow for correction of variations in prescribed dose for patients with differing breast densities who undergo TARGIT-IORT (21). In summary, a better understanding of breast density as a tenet of risk stratification for patients undergoing TARGIT-IORT is an area that warrants further investigation. A means to report breast density more objectively would likely have multiple clinical benefits and improve patient outcomes in this setting.

Patient selection for TARGIT-IORT may be further bolstered using genetic assays such as the Oncotype Dx^®^ scoring system for risk stratification. Schwartzberg et al. reported the results of 184 patients with DCIS and IDC treated with TARGIT-IORT, finding that the 21-gene recurrence score (RS) correlated with 2017 CG guidelines and was a more consistent predictor of distant recurrence risk in TARGIT-IORT patients than suitability grouping (22). In patients with hormone receptor positive, node negative disease, this gene assay is a prognostic tool that has been shown to predict the benefit of chemotherapy and may guide treatment decisions for adjuvant therapy. Furthermore, it is argued that as RS increases, the benefit of radiation on local disease becomes more apparent (23). Although the patients in our cohort did not routinely undergo RS testing, it is possible that future CG guidelines will integrate scoring into patient selection for APBI.

There are limitations to this analysis. It is a retrospective study of a prospective database and subject to the limitations associated with such an analysis. There is bias introduced in the study because patients are first screened by surgeon for TARGIT-IORT suitability rather than by radiation oncology at the time of consultation. Therefore, the registry is limited to those patients who screened as suitable or cautionary status upfront and received TARGIT-IORT. Although over 200 patients were included in this study, few recurrence events occurred, which limits the ability to identify factors associated with recurrence. Additionally, only 17% of all patients treated with IORT received additional WBI, which biases clinical end points such as IBTR rates. A longer follow-up period is needed to fully capture the risk of local recurrence in this cohort, especially given the natural history of ER+ breast cancers (24). However, the longer term data from the TARGIT-A trial suggest that early results remain valid with longer follow-up.

## Data Availability Statement

The raw data supporting the conclusions of this article will be made available by the authors, without undue reservation.

## Ethics Statement

The studies involving human participants were reviewed and approved by The Columbia University IRB (IRB-AAAJ8512). The patients/participants provided their written informed consent to participate in this study.

## Author Contributions

AB and EB have contributed equally to this work and share first authorship. AB organized the database and wrote the first draft of the manuscript. EB also wrote the manuscript. EC contributed to conception and design of the study. SL and GL performed the statistical analysis. All authors contributed to manuscript revision, read, and approved the submitted version.

## Conflict of Interest

The authors declare that the research was conducted in the absence of any commercial or financial relationships that could be construed as a potential conflict of interest.

## Publisher’s Note

All claims expressed in this article are solely those of the authors and do not necessarily represent those of their affiliated organizations, or those of the publisher, the editors and the reviewers. Any product that may be evaluated in this article, or claim that may be made by its manufacturer, is not guaranteed or endorsed by the publisher.
