# Supplementary material for: Targeted Intraoperative Radiotherapy (TARGIT-IORT) for Early-Stage Invasive Breast Cancer: A Single Institution Experience
Source: Front Oncol. 2022 Jun 29;12:788213. doi: 10.3389/fonc.2022.788213 (PMC9277011; doi:10.3389/fonc.2022.788213)
Supplement: Supplementary file 1 [file Presentation_1.pptx]

## Slide 1
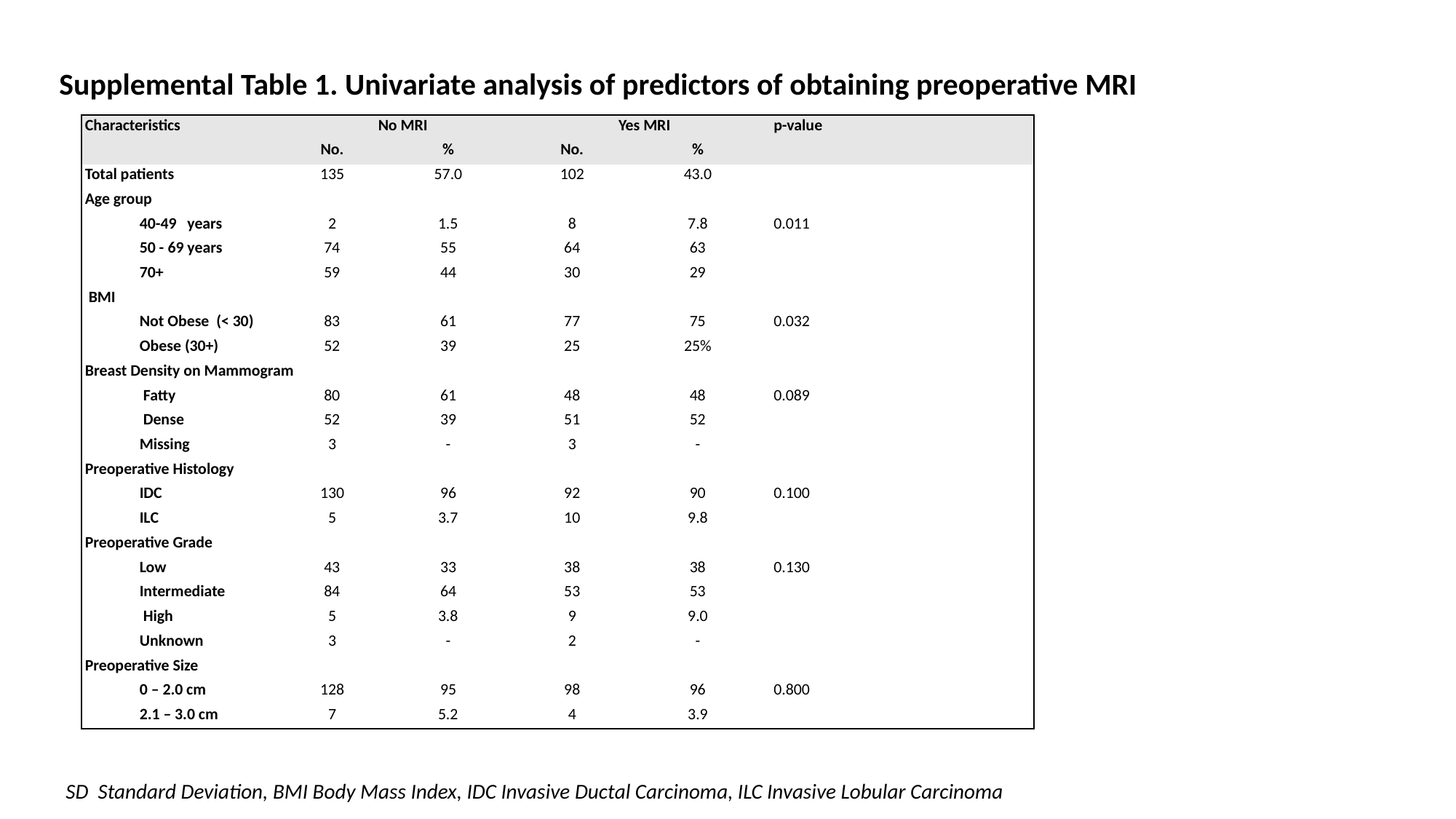

Supplemental Table 1. Univariate analysis of predictors of obtaining preoperative MRI
| Characteristics | No MRI | | Yes MRI | | p-value |
| --- | --- | --- | --- | --- | --- |
| | No. | % | No. | % | |
| Total patients | 135 | 57.0 | 102 | 43.0 | |
| Age group | | | | | |
| 40-49 years | 2 | 1.5 | 8 | 7.8 | 0.011 |
| 50 - 69 years | 74 | 55 | 64 | 63 | |
| 70+ | 59 | 44 | 30 | 29 | |
| BMI | | | | | |
| Not Obese (< 30) | 83 | 61 | 77 | 75 | 0.032 |
| Obese (30+) | 52 | 39 | 25 | 25% | |
| Breast Density on Mammogram | | | | | |
| Fatty | 80 | 61 | 48 | 48 | 0.089 |
| Dense | 52 | 39 | 51 | 52 | |
| Missing | 3 | - | 3 | - | |
| Preoperative Histology | | | | | |
| IDC | 130 | 96 | 92 | 90 | 0.100 |
| ILC | 5 | 3.7 | 10 | 9.8 | |
| Preoperative Grade | | | | | |
| Low | 43 | 33 | 38 | 38 | 0.130 |
| Intermediate | 84 | 64 | 53 | 53 | |
| High | 5 | 3.8 | 9 | 9.0 | |
| Unknown | 3 | - | 2 | - | |
| Preoperative Size | | | | | |
| 0 – 2.0 cm | 128 | 95 | 98 | 96 | 0.800 |
| 2.1 – 3.0 cm | 7 | 5.2 | 4 | 3.9 | |
SD Standard Deviation, BMI Body Mass Index, IDC Invasive Ductal Carcinoma, ILC Invasive Lobular Carcinoma

## Slide 2
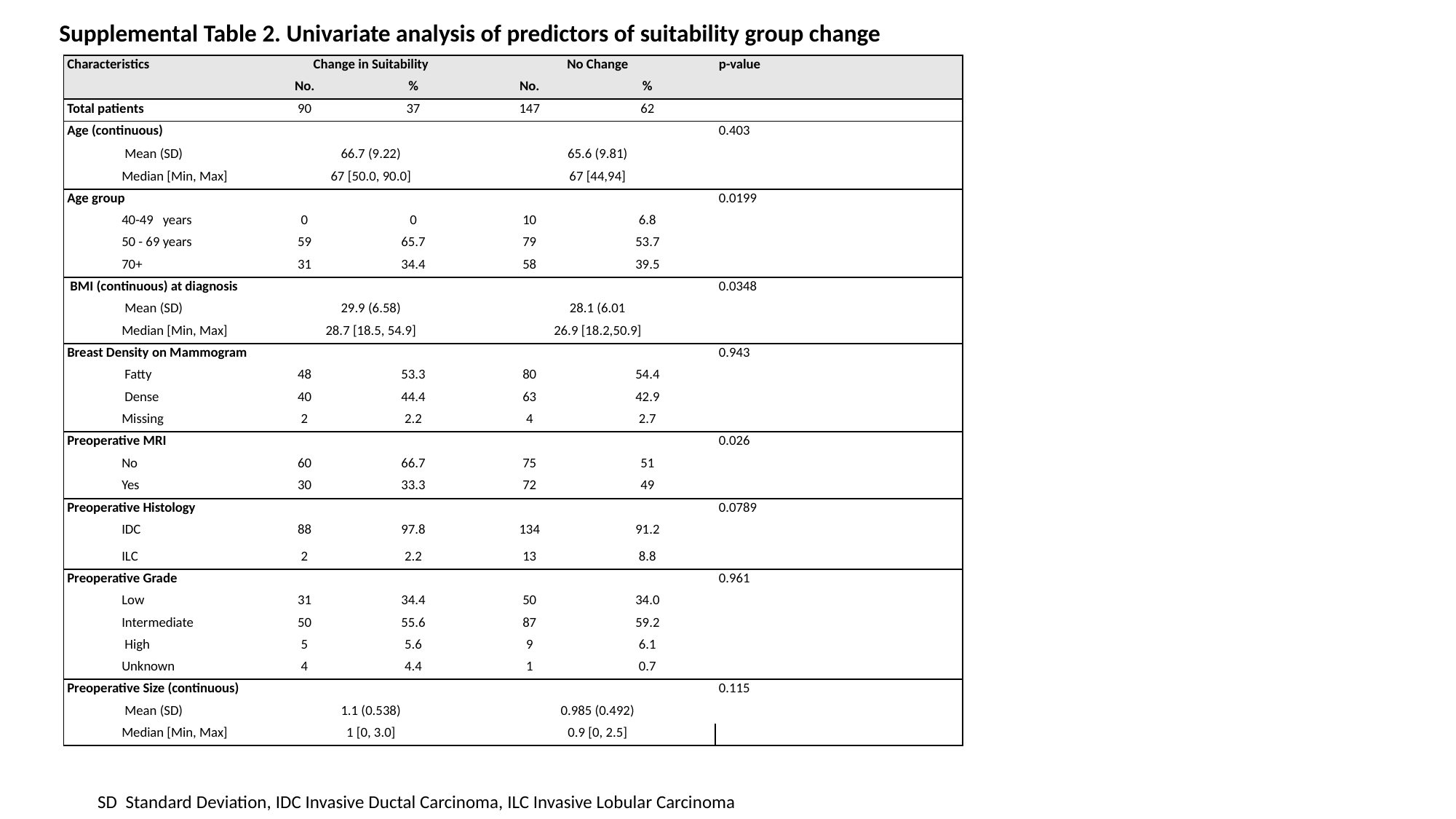

Supplemental Table 2. Univariate analysis of predictors of suitability group change
| Characteristics | Change in Suitability | | No Change | | p-value |
| --- | --- | --- | --- | --- | --- |
| | No. | % | No. | % | |
| Total patients | 90 | 37 | 147 | 62 | |
| Age (continuous) | | | | | 0.403 |
| Mean (SD) | 66.7 (9.22) | | 65.6 (9.81) | | |
| Median [Min, Max] | 67 [50.0, 90.0] | | 67 [44,94] | | |
| Age group | | | | | 0.0199 |
| 40-49 years | 0 | 0 | 10 | 6.8 | |
| 50 - 69 years | 59 | 65.7 | 79 | 53.7 | |
| 70+ | 31 | 34.4 | 58 | 39.5 | |
| BMI (continuous) at diagnosis | | | | | 0.0348 |
| Mean (SD) | 29.9 (6.58) | | 28.1 (6.01 | | |
| Median [Min, Max] | 28.7 [18.5, 54.9] | | 26.9 [18.2,50.9] | | |
| Breast Density on Mammogram | | | | | 0.943 |
| Fatty | 48 | 53.3 | 80 | 54.4 | |
| Dense | 40 | 44.4 | 63 | 42.9 | |
| Missing | 2 | 2.2 | 4 | 2.7 | |
| Preoperative MRI | | | | | 0.026 |
| No | 60 | 66.7 | 75 | 51 | |
| Yes | 30 | 33.3 | 72 | 49 | |
| Preoperative Histology | | | | | 0.0789 |
| IDC | 88 | 97.8 | 134 | 91.2 | |
| ILC | 2 | 2.2 | 13 | 8.8 | |
| Preoperative Grade | | | | | 0.961 |
| Low | 31 | 34.4 | 50 | 34.0 | |
| Intermediate | 50 | 55.6 | 87 | 59.2 | |
| High | 5 | 5.6 | 9 | 6.1 | |
| Unknown | 4 | 4.4 | 1 | 0.7 | |
| Preoperative Size (continuous) | | | | | 0.115 |
| Mean (SD) | 1.1 (0.538) | | 0.985 (0.492) | | |
| Median [Min, Max] | 1 [0, 3.0] | | 0.9 [0, 2.5] | | |
SD Standard Deviation, IDC Invasive Ductal Carcinoma, ILC Invasive Lobular Carcinoma
